# The Influence of Tool Wear on the Mechanical Performance of AA6061-T6 Refill Friction Stir Spot Welds

**DOI:** 10.3390/ma14237252

**Published:** 2021-11-27

**Authors:** Willian S. de Carvalho, Maura C. Vioreanu, Maxime R. A. Lutz, Gonçalo P. Cipriano, Sergio T. Amancio-Filho

**Affiliations:** 1 BMK Endowed Professorship for Aviation, Institute of Materials Science, Joining and Forming, Graz University of Technology—TU Graz, Kopernikusgasse 24/1, 8010 Graz, Austria; willian.carvalho@tugraz.at (W.S.d.C.); gfpcipriano@gmail.com (G.P.C.); 2 Department of Steel Construction and Structural Mechanics, Polytechnic University of Timișoara—UPT, Ioan Curea 1, 300224 Timișoara, Romania; vioreanum@yahoo.com; 3 Sciences et Génie des Matériaux, Institut National des Sciences Appliquées de Lyon—INSA Lyon, 7 Avenue Jean Capelle, 69100 Villeurbanne, France; maxime.lutz78@gmail.com

**Keywords:** refill friction stir spot welding, aluminum welding, aluminum alloy 6061-T6, tool wear, spot welding, process development

## Abstract

The Refill Friction Stir Spot Welding (RFSSW) process—an alternative solid-state joining technology—has gained momentum in the last decade for the welding of aluminum and magnesium alloys. Previous studies have addressed the influence of the RFSSW process on the microstructural and mechanical properties of the AA6061-T6 alloy. However, there is a lack of knowledge on how the tool wear influences the welding mechanical behavior for this alloy. The present work intended to evaluate and understand the influence of RFSSW tool wear on the mechanical performance of AA6061-T6 welds. Firstly, the welding parameters were optimized through the Designing of Experiments (DoE), to maximize the obtained ultimate lap shear force (ULSF) response. Following the statistical analysis, an optimized condition was found that reached a ULSF of 8.45 ± 0.08 kN. Secondly, the optimized set of welding parameters were applied to evaluate the wear undergone by the tool. The loss of worn-out material was systematically investigated by digital microscopy and the assessment of tool weight loss. Tool-wear-related microstructural and local mechanical property changes were assessed and compared with the yielded ULSF, and showed a correlation. Further investigations demonstrated the influence of tool wear on the height of the hook, which was located at the interface between the welded plates and, consequently, its effects on the observed fracture mechanisms and ULSF. These results support the understanding of tool wear mechanisms and helped to evaluate the tool lifespan for the selected commercial RFSSW tool which is used for aluminum alloys.

## 1. Introduction

**Figure 1:**
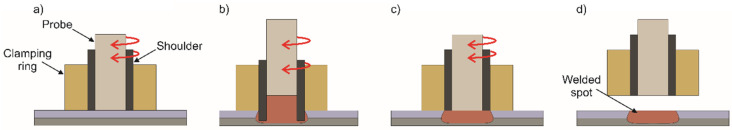
Schematics of RFSSW stages for the shoulder-plunge variant: (**a**) clamping and tool rotation, (**b**) plunging stage—shoulder plunge and probe retraction, (**c**) refilling stage—parts return to surface level and (**d**) tool retraction.

Refill Friction Stir Spot Welding (RFSSW) is a friction-based welding process which has been developed for the welding of metals [1], thermoplastics [2], and thermoplastic composites [3] that has the potential to replace Resistance Spot Welding (RSW) and Self-Piercing Riveting (SPR) techniques in transportation applications [4,5]. The process is performed by using a tool with three non-consumable, independent cylindrical and concentric moving parts—a stationary clamping ring, a rotating probe and a shoulder—as shown in Figure 1. Initially, the clamping ring holds the overlapped welding pieces tightly together against a backing element (a bar, plate or cylinder), preventing the escape of plasticized material during the process. At the same time, the probe and shoulder start to rotate, with the same rotational speed and in the same direction, which generates heat through the friction, and hence plasticizes the material from the upper sheet surface (Figure 1a). Secondly, the shoulder plunges into the work material and the probe moves upwards to create a cavity inside the tool; the rotating shoulder introduces plastic deformation and generates frictional heating. As a result, a volume of the plasticized material is stirred by the shoulder and, due to the material flow, the cavity that was created by the rising probe is filled (Figure 1b). When the desired plunge depth is reached, the shoulder can remain rotating in this position or brought to a halt. In the third step, both the probe and the shoulder return to the original, horizontal level of the top upper sheet surface, forcing the plasticized material that is entrapped inside the tool cavity to refill the keyhole that is left by the shoulder (Figure 1c). In the final step, the clamping pressure is released, and the complete welding tool is withdrawn, thus leaving the weld without a keyhole, and allowing the removal of the welded working material (Figure 1d) [1,5,6]. In addition, a pin-plunge process variant is also possible, however, it is often stated in the literature that the shoulder-plunge technique produces stronger welds [7,8,9].

The RFSSW process has been applied to several aluminum alloys [5,9,10,11], magnesium alloys [12], and also for the joining of dissimilar combinations of materials, such as aluminum to magnesium [13,14], aluminum to steel [15,16,17], and magnesium to steel [12,18], as well as thermoplastics to composites [19] and to nanocomposites [20]. However, this process is still not widely applied in the transportation industries, due to its current state-of-the art, which include a lower technical readiness level and a higher costs-per-produced-spot, when compared to RSW and SPR [21].

As shown in Figure 1, the shoulder and probe are rotating parts and, therefore, they must have small gaps between them—with precise dimensions and tolerance—allowing for their independent movement. Consequently, during the process, those regions are continuously filled with the plasticized material from the workpieces. On one hand, this material operates as a thin protective layer (which is connected to the tool surface by diffusion) that prevents frictional damage between the tool parts; on the other hand, it also causes abrasive tool wear, due to the relative motion of this friction-producing layer [22]. There are only a few studies [22,23,24,25] that have been related to the major influence of the effects of tool wear on the mechanical performances of welds. These works indicate that a considerable decrease in the quasi-static weld strength is expected once the tool is worn out [23]. To prevent this issue, and to avoid premature joint failure, the tool must be replaced after performing a certain number of welds (normally a few hundred for aluminum alloys), increasing the cost of the process, and thereby hindering its industrial application.

The present study aimed to evaluate the effect of this process on tool wear, and its influence on the quasi-static mechanical performance of 6061-T6 aluminum alloy (AA) spot welds. Prior to this investigation, an optimized set of welding parameters were determined, through a combination of the designing of experiments (DoE) with one-factor-at-a-time (OFAT) approaches. In order to assess these effects and correlations, the spot welds’ microstructures and their local mechanical properties were analyzed by optical microscopy and Vickers microhardness tests, respectively. Tool wear was investigated along the welding of up to 2500 spots by digital optical microscopy and the weight loss of the tool components, to determine its influence on the mechanical performance and microstructural features of selected welded specimens. Finally, the analyses of the fracture modes that were observed for the tested welds were reported. The results of this work helped to elucidate tool wear behavior—in respect to microstructural changes and mechanical performance—for the first time in AA6061-T6 RFSS welds.

## 2. Materials and Methods

### 2.1. Base Materials and Welding Equipment

1.5 mm AA6061-T6 rolled sheets (AMAG, Braunau am Inn, Austria) were used in this study. The chemical composition, which was provided by the manufacturer, and the mechanical properties that were experimentally obtained for this material are listed in Table 1. These properties are in accordance with the typical values for this alloy [26].

**Figure 2:**
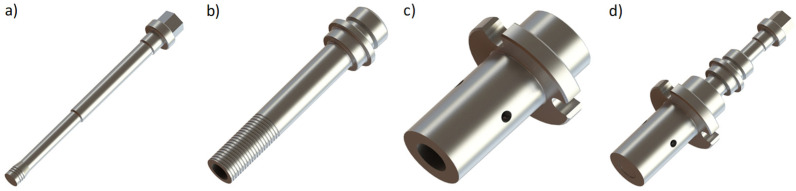
RFSSW tool parts: (**a**) probe—Ø6 mm, (**b**) shoulder—Ø9 mm, (**c**) clamping ring—Ø18 mm and (**d**) assembled tool.

The welds were produced in an RPS 100 RFSSW apparatus (Harms-Wende, Hamburg, Germany), with a standard welding tool composed of H13 tool steel alloy [27], which was provided by the manufacturer and schematically represented in Figure 2. The external diameters of the clamping ring, shoulder and probe tool components were 18, 9 and 6 mm, respectively, whereby the shoulder had a thread groove profile (Figure 2b). The shoulder plunge process variant was applied for all welds, with a constant tool plunge time and refill time of 1 s. Moreover, the tool was installed in the welding equipment with the aid of a dial gauge, in order to keep the misalignment of its main axis below a tolerance of 0.05 mm at the end of the tool and, therefore, minimizing the contact and the consequent, undesired abrasion between the tool components.

### 2.2. Mechanical Testing and Optimization of Welding Parameters

The welding sample geometry which was used in the present work for the optimization of the parameters, as well as the tool wear analysis, was an overlap configuration between two parts, with a 105 × 45 × 1.5 mm and a 35 mm overlap length, in accordance with ISO 14273 [28]. The quasi-static lap shear tests were performed at room temperature, with a crosshead speed of 2 mm/min in a Zwick universal testing machine (Zwick/Roell Group, Ulm, Germany) which was equipped with a 100 kN load cell. 

The optimization methodology consisted of three different phases which were conducted in the following order: DoE, ANOVA, and one-factor-at-time (OFAT) methods. The DoE is an effective and proven method [29,30,31,32,33], used to evaluate the process variables’ effects on a desired response in RFSSW. In this study, a Box-Behnken Design (BBD) model which had three factors (process parameters)—plunge depth (PD), rotational speed (RS), and dwell time (DT)—with three levels each, was used to correlate the ultimate lap shear force (ULSF) response with the welding parameters. Table 2 compiles the process parameter sets that were used. The ranges (levels) used in the BBD experiments were selected based on the preliminary process parameter screening investigation.

In general, the BBD for the three factors and three levels suggested 13 parameter combinations, including the center point. In order to increase the model’s reliability, five additional center point replicas were used for the present study. Therefore, 18 joints were produced and tested in this first optimization step. The welds were randomly produced, and the parameters that were used are presented in Table 3.

After testing the welded samples, the ULSF was used as the investigated response and ANOVA was applied to evaluate the model’s fitting, and to determine the statistically significant factors (main and interactions) [34,35]. The confidence level was set at 95% (i.e., α = 0.05), to assess the *p*-value test results. 

An OFAT approach was used after the initial DoE, to deepen the investigation and to evaluate the individual effect of each parameter analyzed. With this method, the values of two parameters were kept constant, and one was changed, starting from a central point which was chosen as the parameter set with the highest ULSF, from the BBD analysis. This methodology was used to ensure that a global—and not local—peak was found for the optimization process, and that the maximum ULSF was obtained.

### 2.3. Microstructural Analysis, Local Mechanical Properties, and Thermal Characterization

The microstructural analysis of selected welded samples was performed by cutting the specimens, producing cross-sections near the center of the weld, and subsequently preparing the exposed surface by a standard metallographic preparation. Etching was carried out with Weck’s reagent solution (130 mL water, 4 g KMnO4 and 1 g NaOH) for 9 s. In addition, the microstructure of the material was examined by optical microscopy, by applying an Axio Observer 7 (Zeiss, Jena, Germany). Finally, the mean grain sizes of the different welding zones were determined, in accordance with the ASTM E112 standard, by applying the circular test method [36]. Several circular areas were selected in random regions, in order to mitigate any bias.

Vickers microhardness measurements were performed to evaluate the welds’ local mechanical properties on the metallographic specimens, in accordance with the ASTM E384-11 standard [37]. This had the objective to determine the transition areas that were between the different welding zones. These measurements were carried out using a load of 200 g, applied for 15 s, with a spacing of 0.5 mm between two adjacent indentations.

**Figure 3:**
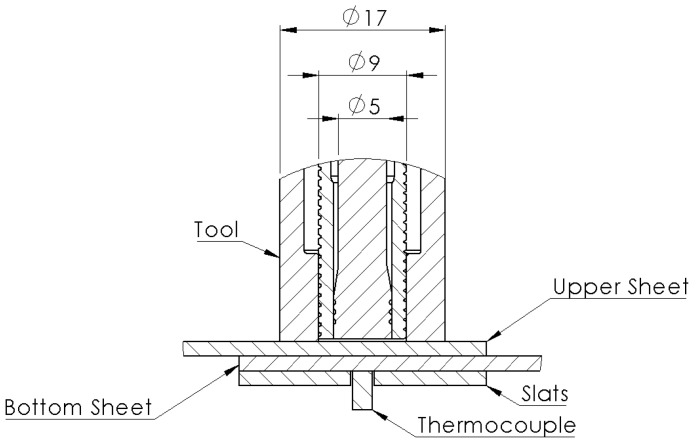
Set-up used for temperature measurements.

The process temperature was recorded at specific welding cycles to assess the process frictional heat input. Measuring the temperature at the volumetric center of the spot was not possible during the process, due to the rotation and displacement of the tool. Therefore, a K-type thermocouple was positioned underneath the welding spot area, on the bottom surface of the lower plate, 1 mm away from the spot center. To prevent the thermocouple from being destroyed under the clamping pressure of the equipment, two supporting slats were placed beside it, leaving a gap for the thermocouple to be safely placed, as shown in Figure 3.

### 2.4. Tool Wear Analysis

The tool wear analysis involved the observation of changes which occurred on the shoulder thread groove profile, after several welding cycles. For this purpose, a Keyence VHX6000 digital microscope (Keyence, Osaka, Japan) was used to evaluate this morphological features and the macro changes in its contour. Moreover, the weight loss of the shoulder was quantified by using a high precision scale, with a readability of 1 mg. Before these analyses, the tool was cleaned in a solution of 1 g NaOH, in 150 mL of water, at 40 °C, for one hour, to remove the aluminum residue attached to its surface as a result of the welding process.

## 3. Results and Discussion

### 3.1. Process Parameters Optimization

One important issue in the optimization process is in finding the contribution percentage of each welding parameter that affects the joint strength. By applying ANOVA, a reduced statistical model was obtained, and the terms which were considered statistically significant were identified. Therefore, only terms with a *p*-value lower than 0.05 were considered. Table 4 summarizes those terms and their respective *p*-values.

**Figure 4:**
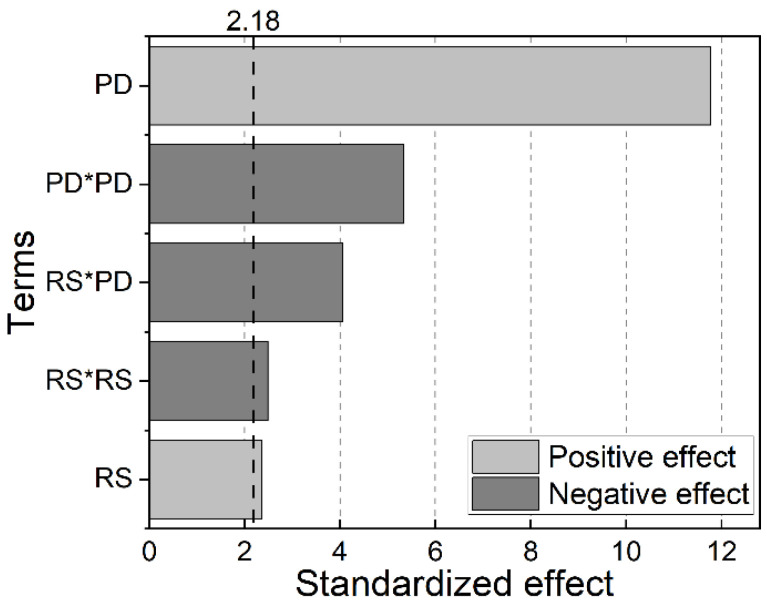
Pareto chart of the ANOVA performed on ULSF response regarding the significant factors and interactions effects.

The analysis of the obtained *p*-values shows that, predominantly, two main parameters and three interactions were statistically relevant for ULSF: the plunge depth (PD) and rotational speed (RS) main effects; the PD*PD and RS*RS second-order interaction effects, as well as the two-way interaction effect of RS*PD. A Pareto chart can be plotted to graphically illustrate the magnitude of each effect and its contribution to ULSF, therefore facilitating the discussion and comprehension of the obtained results. This chart is presented in Figure 4.

In the present Pareto chart, the length of each bar is proportional to the standardized effect of each parameter or interaction, i.e., the effects of the analyzed parameters and interactions are divided by their standard error; they are presented in decreasing order of relevance. The vertical dashed line represents the relevance threshold, which delimits the statically significant parameters at a 95% confidence level; therefore, any bar that crosses this line corresponds to an effect that is statistically representative for the considered ULSF response.

As one can observe, the PD parameter is the single main effect with the strongest influence on the weld quasi-static mechanical performance and a presented positive value (synergic behavior, see Equation (1)). Consequently, a minor change in its value would strongly impact the ULSF of the produced weld. Similarly, the RS also presented a significant and synergic estimated effect, but of a smaller proportion. In short, the presented results show that ULSF tends to increase with higher PD and RS values. In contrast to both effects, DT did not show statistical significance in this study; hence, the ULSF does not appear to depend on the DT process parameter when its value is between zero and two seconds for the analyzed conditions.

In addition to the obtained *p*-values, the statistical analysis also provided a reduced regression model for the ULSF, as well as important indices that can be used to assess the explanatory power of the equation. The reduced model, obtained via backward elimination with α = 0.05, is presented in Equation (1), where the response ULSF is a function of the analyzed welding parameters, PD and RS:

ULSF [kN] = −15.69 + 0.004651*RS + 20.55*PD − 0.00000046*RS² − 4.540*PD² − 0.001695*RS*PD(1)

**Figure 5:**
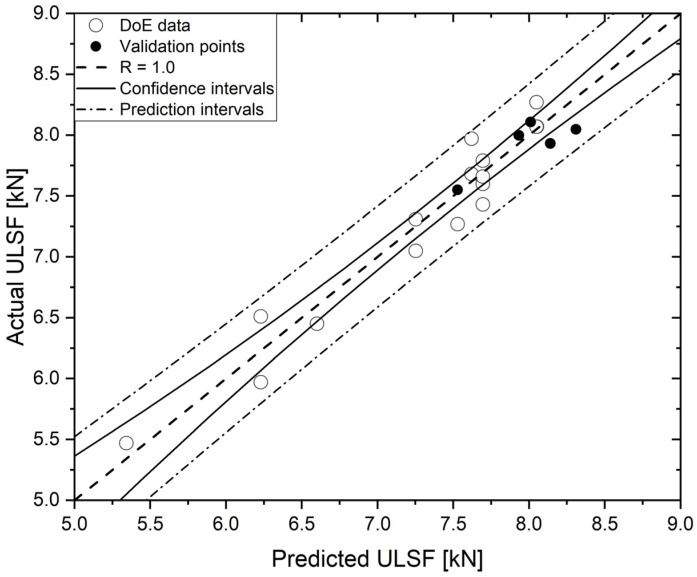
Predicted versus actual diagram for the ULSF model.

The experimental ULSF values that were measured are shown in comparison with the model predictions in Figure 5. Additional validation points were randomly produced, and their results are also displayed.

As one can see, the graph shows that all the design points fall within the model prediction interval that comprehends 95% of confidence—this indicates a good correlation with the experimental data. In addition, the adjusted and predicted R-sq which were obtained for the model were, respectively, 91.93% and 80.64%, with a standard error (S) of 0.22 kN. As explained by Myers, Montgomery and Anderson-Cook [38], if the difference between the adjusted and predicted R-sq is less than 20%, the model fits the data, and can then be used to interpolate other points. Considering the complexity levels of the phenomena which are involved in solid-state welding techniques [39], the results that were achieved in this study were considered satisfactory.

**Figure 6:**
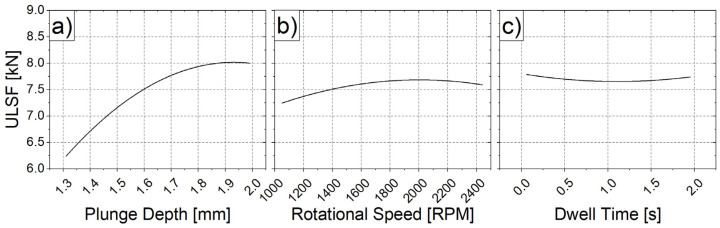
Main effects for ULSF (**a**) plunge depth, (**b**) rotational speed, and (**c**) dwell time.

Considering only the main effects for the present condition, the ideal welding parameter set for maximum ULSF would be high values for both the PD and RS, with the value of DT being irrelevant, from a performance point of view. From a process standpoint, the lower the DT value, the shorter the time that is consumed per spot that is produced. Therefore, setting the DT to 0 s can lead to potential cost savings for this process. Figure 6 shows the estimated ULSF response for each effect. In each plot, the variables are varied from their lowest to their highest values. All other variables, beside the one being varied, are kept constant to their central values. 

The plots presented in Figure 6 partially confirmed the expected trends. By analyzing Figure 6a, it is possible to state that increasing the value of PD would result in stronger joints, at least until 2 mm. In contrast, Figure 6b shows that values which are close to the RS limits (minimum and maximum) lead to weaker welds. An increment of RS from 1000 to 1800 RPM increases the ULSF, since it increases the welding temperature and leads to a better plasticization of the material [40]. The turning point presented around 1800 RPM is associated with a decrease in the heat input, which is a function of torque. When the RS becomes too high, material viscosity—and the observed torque—decreases, reducing the heat generation during the process and, consequently, decreasing the material plasticization, as well as hindering a proper material flow and intermixing. Therefore, a lower ULSF is reached for a higher RS [41].

In terms of DT, Figure 6c presents an almost constant behavior for the curve, independently of its value (graph slope ≈ 0). Hence, two different welds which are produced with an equal PD and RS, but a different DT, tend to have a similar ULSF response. As it can be seen, the set of welding parameters that were determined by this method (a PD of 2 mm; an RS of 1750 RPM; and a DT of 0 s), is capable of producing the strongest welded joint (8.3 kN, Table 3).

**Figure 7:**
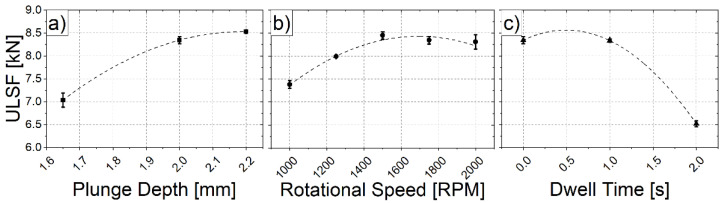
Obtained results for the OFAT analysis with varying (**a**) plunge depth, (**b**) rotational speed, and (**c**) dwell time.

To investigate each parameter’s individual effects, and to ensure that the maximum ULSF peak was reached by the indicated BBD parameters which were set, an OFAT methodology was applied. The results were plotted in the charts presented in Figure 7.

Generally speaking, the same behavior observed with the BBD analysis was observed for PD (Figure 7a) and RS (Figure 7b). On the other hand, DT (Figure 7c) appeared to completely differ from the pattern that was previous described—i.e., the effects of the increase of DT on the welds’ mechanical performance were detrimental for the ULSF at the upper limit value (2 s). Cao et al. [42] addressed this behavior for similar AA6061-T6 RFSS welds and showed that an increase in the joining time increased the hook height and resulted in lower weld strength, since the observed fracture mode changed for long joining cycles. This deviation from the results of the BBD can be explained by the lower accuracy that a BBD has for the parameter window extremes, since no experimental parameter combinations which use all the simultaneous maximums have been performed. Furthermore, all the analyzed combinations showed low standard deviation values, which indicate that the RFSSW process was stable and has a high ULSF reproducibility for the AA6061-T6 in the analyzed parameter windows.

Regarding the RS parameter, the graph in Figure 7b shows that 1500 RPM produced welds which were slightly stronger (8.45 ± 0.08 kN) than those produced with 1750 RPM (8.34 ± 0.08 kN) that have been previously recommended by the BBD method. Concerning the PD (Figure 7a), the curve confirms the behavior which was previously stated. Even though the PD of 2.2 mm produced the strongest welded joint, its application was not feasible, since its frictional heat input was excessive, which increased the amount of plasticized material and, as a result, the bottom aluminum sheet was often welded to the supporting sample holder. Therefore, the optimized process parameter set was determined as: a PD of 2 mm; an RS of 1500 RPM; and no DT (0 s). The welds produced with this combination surpassed the results that were obtained with the BBD combination and reached an average of 8.45 ± 0.08 kN, therefore maintaining the observed high reproducibility and reliability.

### 3.2. Tool Wear Evolution and Weld Mechanical Performance Investigation

**Figure 8:**
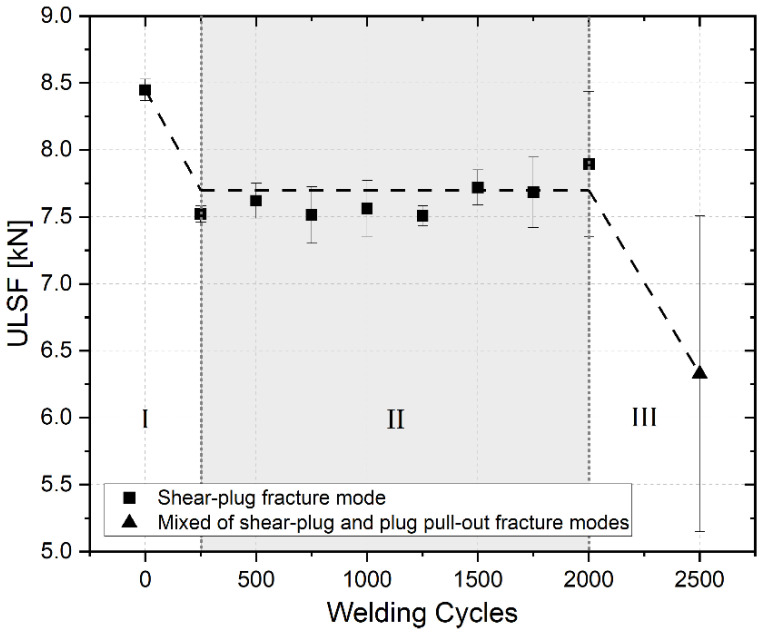
Correlation between ULSF and fracture mode with the number of spots produced.

For the purpose of analyzing the tool lifespan, multiple spots were produced with the same tool, using the optimized parameter set that was discussed in the previous section. Initially, 250 spots were sequentially produced, of which the last six specimens were welded in a lap shear configuration. Three of these lap shear samples were quasi-statically tested and had their fracture mode further investigated. Furthermore, the remaining three samples were prepared for a metallographic analysis. This process was repeated until a number of 2000 spots was reached. Afterwards, the interval was increased to 500 spots to accelerate the process, based on the rather lower ULSF variation that was observed for the previous spot welds. The tool wear analysis was ceased at 2500 spots, because the wear increased the gaps between the tool pieces, which led to a higher accumulation of material between the shoulder and the clamping ring. This aggregation obstructed the tool rotation; therefore, the maximum equipment torque threshold was often reached prematurely, and automatically interrupted the process. The obtained results for the ULSF, as a function of the spot weld number, are presented in Figure 8. The figure also indicates the fracture modes which were observed for each analyzed condition.

Predominantly, the ULSF decreased over the analyzed interval, which indicated that the number of welding cycles negatively affected the mechanical performance of the spots that were produced. The strongest joint was produced when the tool was new, and the weakest was produced after 2500 spots. For a better visualization, the curve was divided into three phases, represented as dashed trend lines in the graph. Furthermore, the graph shows that the calculated standard deviation drastically rose as the number of welded joints increased, and that the observed fracture mode had changed by the end of the investigation.

Initially, the spots which were produced with the new tool reached an average ULSF of 8.45 ± 0.08 kN, as previously presented in the optimization process. As soon as the study started (Phase I), and the number of 250 spots was reached, the curve decreased by approximately 11% and hit a plateau that was close to 7.7 kN. This behavior persisted until 2000 spots (Phase II), with the values remaining relatively steady, and with a low fluctuation. Finally, the ULSF values fell sharply as 2500 spots were reached (Phase III) and, consequently, the lowest ULSF was measured—6.33 ± 1.18 kN.

**Figure 9:**
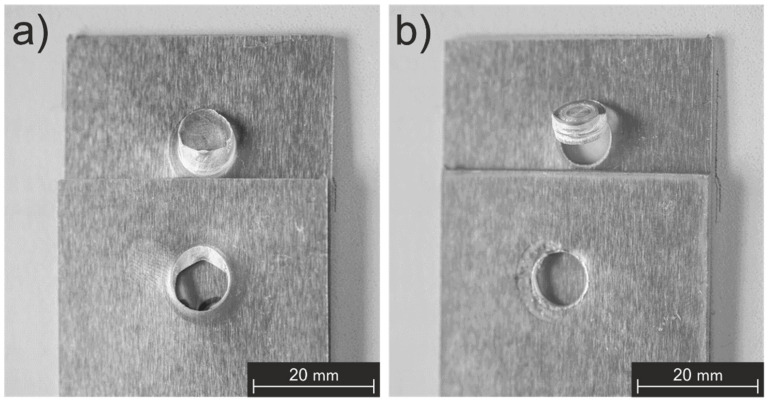
Fracture modes observed after lap shear tests of the welded samples; (**a**) shear-plug fracture mode after 1250 spots and (**b**) plug pull-out fracture mode after 2500 spots.

The fracture modes for all the tested samples were analyzed in an effort to explain the decline in mechanical performance. As shown in Figure 8, only two different modes were observed: shear-plug fracture and plug pull-out fracture [43,44]. The vast majority of the samples demonstrated only the shear-plug fracture mode, with only two of the three samples tested after 2500 spots failing through plug pull-out mode. Figure 9 presents examples of both fracture types.

According to Rosendo et al. [5], the type of fracture mode observed in tested samples can be correlated to their mechanical performance. In other words, the ULSF that is reached by the specimens strongly depends on the path followed by the crack during its propagation. In addition, Chen [45] correlated several weld strengths with their respective fractured surfaces and found that the plug pull-out failure mode results in weaker spots when compared with the spots that failed by the shear-plug mode. Consequently, it is possible to state that the decline that was observed in Figure 8, when 2500 spots were produced, resulted from the change in the fracture mode.

**Figure 10:**
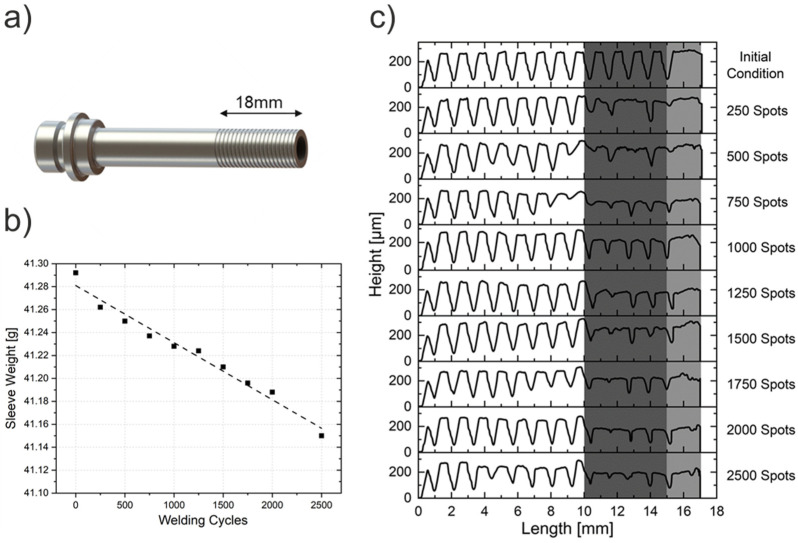
Tool analyses: (**a**) Shoulder geometry with threaded length, (**b**) shoulder material loss, and (**c**) shoulder grooved profile with the advancing of produced spots.

Furthermore, to explain and understand the observed change in the fracture mode behavior, the tool wear was quantified, and the produced welds’ microstructures were analyzed. As previously stated, to allow the motion of the probe and the shoulder, small gaps between all three tool parts are necessary and, during the process, the plasticized material flows into those cavities and causes wear of the tool due to abrasion, which increases as more spots are produced. Moreover, the contact between the shoulder and the material during the plunging stage of the process is also expected to wear out the tool material. In order to analyze and quantify these changes on the shoulder thread groove profile, digital microscopy measurements and the tool material weight loss were investigated. Both the clamping ring and probe were also analyzed, although, in contrast to the shoulder, they did not present any significant change; therefore, they are not discussed in this study. Figure 10 shows the wear evolution of the shoulder profile during the analyzed lifetime and its weight loss.

Figure 10a schematically shows the threaded part of the shoulder profile, with a length of 18 mm, starting from the face that plunges into the material. Regardless of the small percentual variation of approximately 0.34% (or 0.142 g), a linear trend of an increasing shoulder weight loss is shown in Figure 10b, which, in addition to Figure 10c, shows that the shoulder profile clearly lost material as the number of produced spots increased. The damaged region can be divided into two parts, which are represented as dark and light grey areas in Figure 10c. 

**Figure 11:**
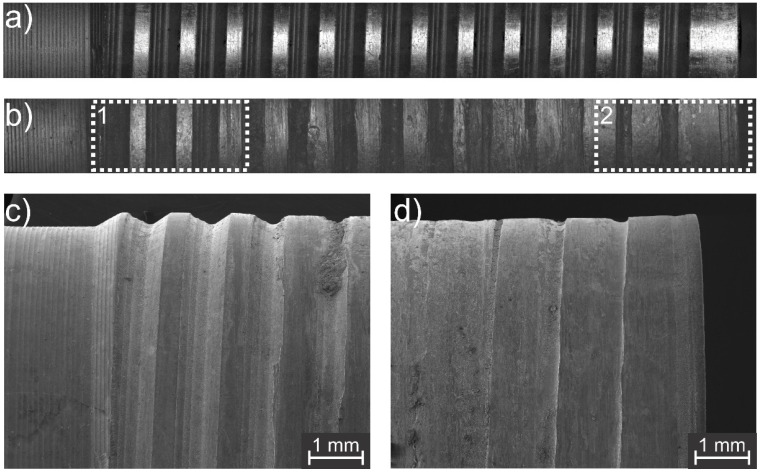
(**a**,**b**) shows an overview of the shoulder groove profile in its initial condition and after 2500 welded spots, respectively; (**c**,**d**) show detailed surface analyses of the undamaged (region 1) and damaged (region 2) tool areas, respectively.

The light grey area in Figure 10c represents the 2 mm plunged into the sheets which wears off when stirring the base metal. In addition, the dark grey region highlights the part of the tool that experiences abrasion from the material overflowing into the gap between the shoulder and the clamping ring. For a better comparison, Figure 11a shows an overview of the shoulder groove profile in its initial condition, and Figure 11b shows the profile after 2500 welded spots. Both images were obtained via digital microscopy. Additional detailed surface analyses by scanning electron microscopy of the worn tool were carried out to better illustrate the tool wear effect. Figure 11c shows details of the undamaged tool regions, and Figure 11d shows the damaged tool regions.

In summary, it is possible to affirm that the shoulder geometry changed, due to the observed tool wear. Therefore, one may conclude that the frictional regime changed with the tool wear evolution, thereby modifying the heat input. Different energy input levels are expected to result in different weld microstructural features; therefore, a microstructural analysis was conducted.

### 3.3. Microstructural Analysis, Temperature Measurements, and Microhardness Analyses

**Figure 12:**
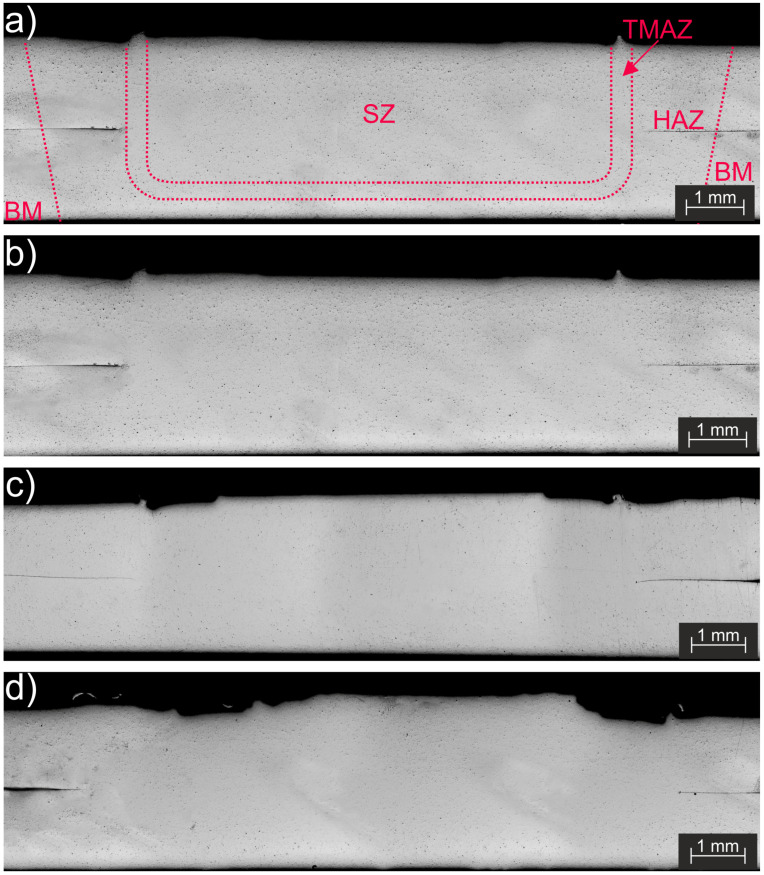
(**a**) Schematic of a typical RFSSW cross-section with its main regions; cross-sections from the center of the spots in different conditions: (**b**) new tool, (**c**) 1250, and (**d**) 2500 spots.

The RFSSW technique, as with any other friction-based welding process, introduces heat and plastic deformation into the weld area, and this consequently modifies the material microstructure. These changes are frequently related to their mechanical properties and fracture behavior. In order to analyze the presence of defects and quantify the tool-wear-related microstructural changes, cross-sections from the center of the spots were analyzed via optical microscopy, and the average grain size was determined for three conditions: new tool, 1250, and 2500 spots. For these analyses, three replicas were prepared and analyzed for each condition. Normally, the microstructure resulting from a RFSSW welding cycle can be classified into four distinct regions, which are typical for friction-based processes that are applied to aluminum alloys [46,47]: the base material (BM); the heat-affected zone (HAZ); the thermo-mechanically affected zone (TMAZ); the stir zone (SZ). Figure 12a schematically shows these microstructural zones within an RFSS weld. Figure 12b–d present cross-section examples from the center of the spots for a new tool, 1250, and 2500 spots.

Figure 12a shows the schematic position and geometry of the four welding zones (BM, HAZ, TMAZ and SZ). Figure 12b–d shows the cross-sections of welds that were produced with a new tool, as well as after 1250 spots and 2500 spots, respectively. The obtained images show that the presence of defects (e.g., voids, lack of bonding or of mixing, incomplete refill, etc.) was not detected in any of the analyzed welds. According to Wang et al. [48], the lack of volume defects indicates that the frictional heat generated by the shoulder and probe was enough to plasticize the material. This demonstrates that the observed tool wear did not reach the critical point to introduce defects inside the analyzed welds. However, as one can see, the surface of the upper sheet after 2500 spots seems to be less smooth than the ones for the initial condition and 1250 spots, indicating that the tool wear influenced the finishing surface of the produced spots.

**Figure 13:**
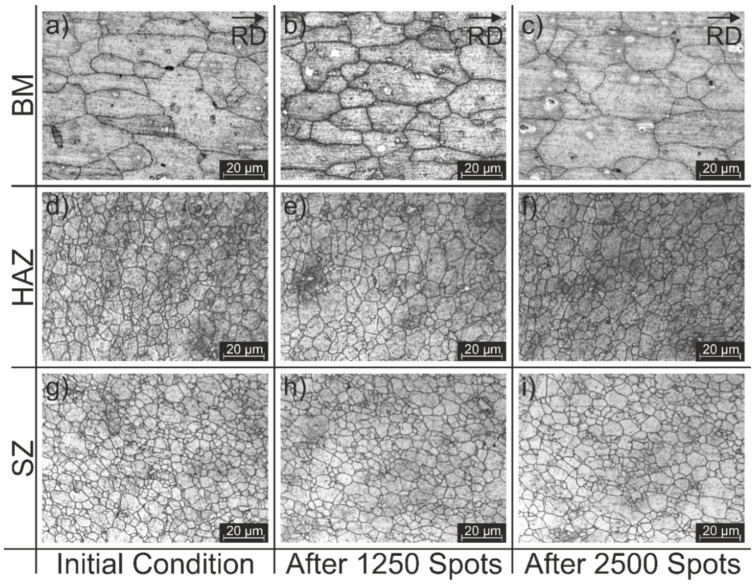
Microstructural aspects of the welding zones which were identified: (**a**–**c**) BM, (**d**–**f**) HAZ, (**g**–**i**) SZ.

To further investigate the influence of the tool wear on the produced spot welds, the microstructures of the different zones were further analyzed for all three specimens. Figure 13 shows detailed micrographs from the microstructural zones identified. The rolling direction (RD) is shown for the base material images.

In general, it is possible to observe that, in the BM, the grains are elongated in the rolling direction of the material. By definition, the HAZ only experiences the thermal welding cycle, and no plastic deformation occurs in this zone during RFSSW. Hence, the microstructure in the HAZ is similar, although slightly coarser when compared to that of the BM. The TMAZ is located at the outside of the shoulder outer radius, and is typically characterized by highly deformed grains in comparison to those in the BM and HAZ. However, in this study, it was not possible to define any clear transitions between the HAZ/TMAZ and the TMAZ/SZ, due to the thinness of the TMAZ region. Attempts to do so resulted in erroneous measures and, therefore, this zone was excluded from the analysis. The SZ presents finer, equiaxed grains, which can be attributed to the occurrence of dynamic recrystallization that was caused by the high strain rate and high temperatures reached during the process [47]. The obtained results for the grain size measurements of the different zones, from the micrographs in Figure 13, are summarized in Table 5.

According to the average grain size, there was no evidence of a particular correlation between the grain size variation and tool wear in the different weld zones; that is, in the different areas, the grain size tended to be similar in the different tool conditions. Therefore, a different grain size distribution was not indicated as the cause of the change in the observed fracture behavior and, consequently, the origin of the decline that was observed in the mechanical performance of the welds.

**Figure 14:**
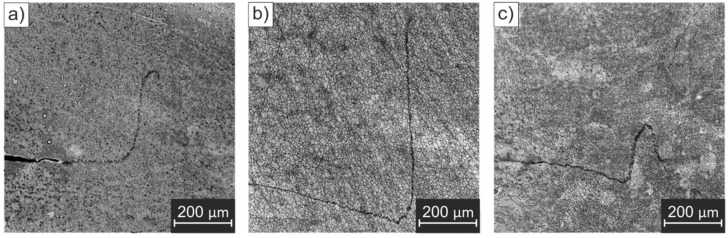
Hook configuration at (**a**) initial condition with a new tool, (**b**) after 1250 spots, and (**c**) after 2500 spots.

**Figure 15:**
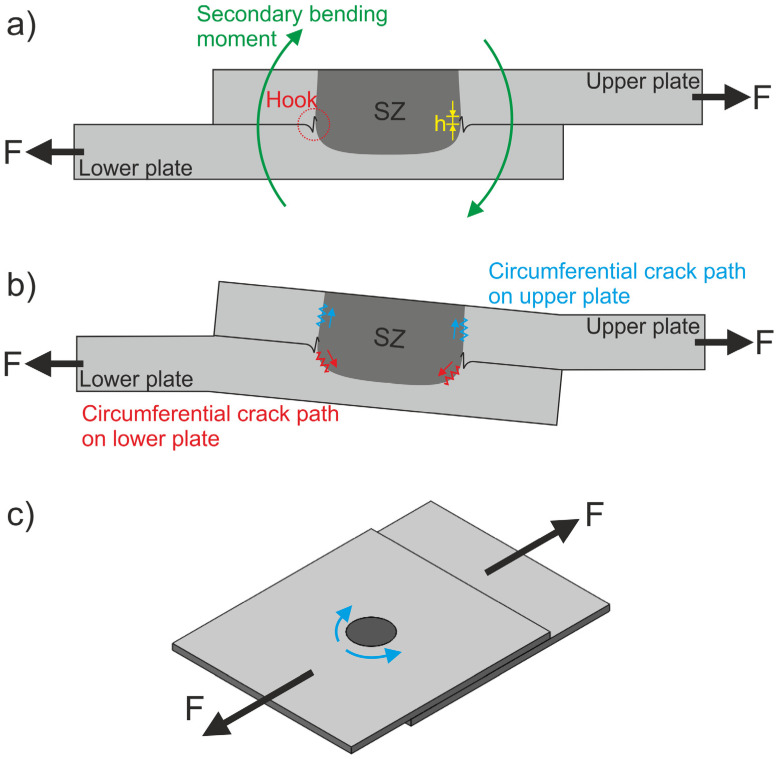
Schematic illustrations of (**a**) the hook location and height, (**b**) fracture development of RFSS welds under quasi-static lap shear test, and (**c**) a representation of circumferential crack propagation around the SZ along the hook.

Rosendo et al. [49] discussed different scenarios to explain the fracture modes in the RFSSW of AA6181-T4 single lap joints; the authors found a correlation between another microstructural feature called the hook. The hook is produced during the refilling stage at the interface between the two sheets, and can act as a nucleation site for cracks, which affects the fracture mode and impacts the weld mechanical properties. The hook is formed due to the material flow that is promoted by the shoulder movement and its rotation during the process. According to Silva et al. [50] and Sun et al. [51], the observed fracture modes are often related to the shape and height of the hook. In other words, the mechanical performance of the spot is strongly related to the hook configuration. The hooks that were observed for a new tool, after 1250 spots, and after 2500 spots are presented in Figure 14. Figure 15a illustrates the hook position on both sides of the SZ.

From the figures, the hook shape that was observed for all three conditions was similar, resembling an S-shape. This behavior was expected, since the PD was kept constant during this study and, as demonstrated in the literature [51], PD is one of the main parameters in control of the hook formation and geometry. As demonstrated by Cao et al. [42], this influence of the PD on the hook shape develops from the plunging stage of the shoulder, which pushes the softened material beneath it downward, squeezing the adjacent material upward. On the other hand, it is possible to see that the hook height clearly changed for each sample, which might explain the differences in their mechanical performance.

Yin et al. [52] stated that the smaller the hook is, the higher that the quasi-static strength of the weld reaches. In their study, they reported that, during lap shear testing, failure initiation occurs at the tip of the hook and, therefore, mechanical properties are strongly influenced by its height. A pronounced vertical displacement, which refers to the ratio between the hook height and the initial sheet thickness, facilitates a failure mechanism under shear loading. In the present case, this appears to be valid as well, since the weld which was produced with a new tool showed a higher ULSF (8.45 ± 0.08 kN) than the weld produced after 1250 spots (7.51 ± 0.07 kN). However, the smallest hook was observed after 2500 spots, which also provided the lowest mechanical performance, an unforeseen result. In order to explain this difference, it is important to analyze the observed fracture modes which are presented in Figure 9 and to understand how the hook can influence the observed fracture mechanisms. Figure 15 shows, schematically, the role of the hook in crack propagation and the mechanical behavior of single lap joints.

Figure 15a shows the hook’s location and how its height can be specified by considering the interface between the welded plates. During a quasi-static lap shear test, the eccentricity of the load line between the upper and lower sheets promotes secondary bending, which generates a tri-axial stress state, and can lead to crack initiation and propagation [53]. In the shear-plug fracture mode, the crack propagates toward the surface, before propagating at the weld’s circumference, as shown by the blue arrows in Figure 15b. On the other hand, in the plug pull-out failure mode, the crack propagates preferably to the center of the weld, and around the SZ at the interface between the two sheets, as represented by the red arrows in Figure 15b [49]. After the crack’s nucleation in the hook region and its growth through the sheet, the crack propagates around the SZ, as represented by Figure 15c, which leads to the development of these failure modes. Due to these crack propagation paths, the shear-plug fracture failure mode is reported to achieve higher strength values than the plug pull-out failure mode—which, in this case, occurred in the lower sheet. Therefore, even though the hook height is smaller, the mechanical performance of the joint after 2500 spot welds was lower due to the change in the fracture mode. This behavior indicates that there is a critical hook height for which the type of fracture changes. Since the welding parameters were kept constant during the study, the tool wear must have been the variable that influenced this modification. In comparison to Figure 14a, Figure 14b indicates that the frictional heat input was higher in the joints produced with this condition—after 1250 spots—and, consequently, that the level of plasticization reached by the aluminum was also superior, which increased the height of the hook. Likewise, Figure 14c indicates that this energy input was lower.

**Figure 16:**
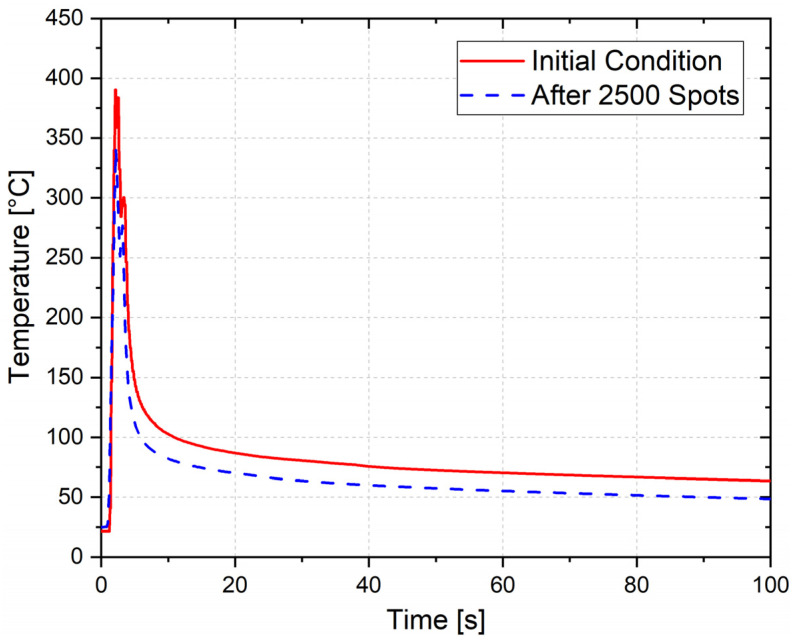
Evolution of the temperature underneath the spot region during the welding process.

Different energy levels should have resulted in visual differences in the material microstructure; however, as previously stated, the average grain size measured in the HAZ and SZ for the different conditions showed similar values. This analysis illustrates the material structure in the micro scale, and does not show the differences in the macro scenario. Therefore, the temperature was measured for the initial condition—with a new tool—and after 2500 spots, to determine if any changes could be observed in the heat generation. Following that, these results were compared against the evaluated microhardness profiles. The obtained temperature curves for the new tool and after 2500 spots are presented in Figure 16. 

The curves show that, although the welding cycle is very short—with a steep slope—the energy input is sufficient to produce high temperatures (≈340–390 °C). For the new tool, the peak temperature of 390 ± 30 °C is reached in about 0.9 s. Since the dwell time of the tool inside the material was set to zero, as outlined in the process parameter set, as soon as the tool reached its maximum plunge depth, it retracted upwards until it reached the material surface level. Hence, the cooling of the material started immediately, and the temperature decreased to 100 °C in approximately 10 s. After 2500 spots, the maximum temperature that was measured was just 341 ± 25 °C. As described in Section 2.3, the temperature measurements were done on the lower metallic sheet surface, 1 mm away from the spot center. Therefore, one can expect that the temperature at the core of the spot must be higher.

**Figure 17:**
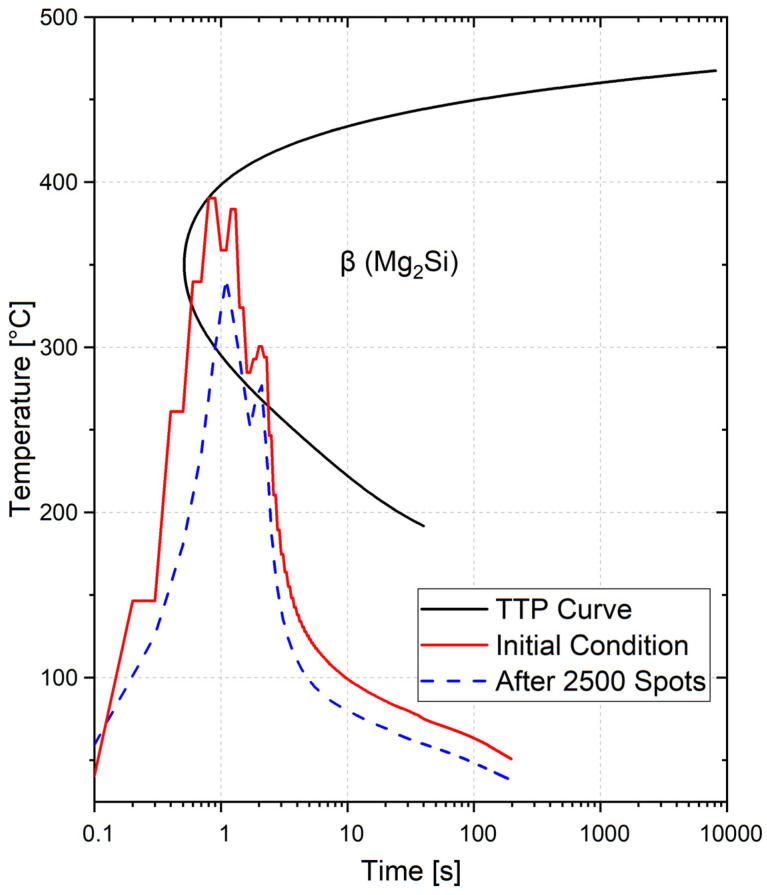
The TTP Vickers hardness C-diagram for AA6061. Adapted from [55].

These curves already show evidence of the process temperature that was reached during the welding cycle. Nevertheless, the complete alloy behavior, regarding the temperature and time, must be well-known in order to understand the heat input effects on the material. For this purpose, Figure 17 shows the Time-Temperature-Property (TTP) Vickers hardness C-diagram for the AA6061 alloy, and the temperature curves for using both the new tool and after 2500 spots. The TTP diagrams (for heat-treatable alloys) describe the time-related changes in strength (represented by hardness), related to equilibrium precipitates [54].

**Figure 18:**
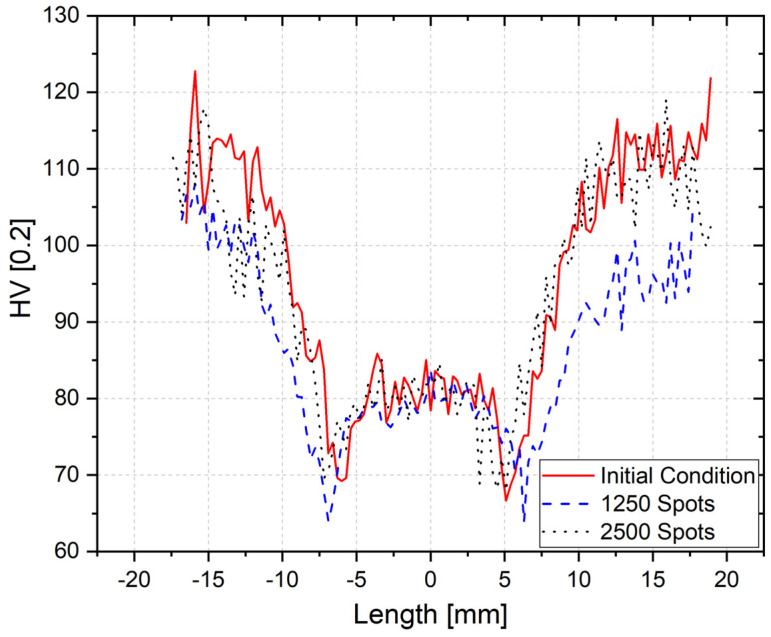
Microhardness profiles from a new tool, after 1250 spots, and after 2500 spots measured at the center of the upper sheet—0.75 mm from the surface.

It is well known that the mechanical properties of precipitation-hardenable, aluminum alloys, such as AA6061, depend on the level of precipitation, as well as the changes in precipitate features that occur during and after (cooling phase) welding. According to Dolan and Robinson [55], the AA6061 material has a high quench sensitivity, and it has a critical temperature range, between 220 °C and 440 °C [56]. Since the main precipitation phase, which occurs before welding, is the coherent β-phase, if a heat treatment or welding process profile crosses the TTP curve (Figure 17), this will lead to the material softening by the dissolution of this phase and the introduction of β (Mg_2_Si) precipitates [56]. Considering the temperature measurement presented in the Figure 16, for a temperature of 390 °C, the softening already starts to occur after one second. The fact that there is a heat gradient in the aluminum sheets during the welding process must also lead to a gradient in the mechanical properties throughout the welded volume. As previous stated, the grain size measurement illustrates the material status in the micro scale, however no variations were observed for the analyzed conditions. Therefore, in order to confirm that the tool wear affected the RFSSW heat input, microhardness profiles were measured in the joints which were produced with a new tool, with the aim of inferring if a local softening had taken place after 1250 spots and after 2500 spots. Figure 18 shows the obtained microhardness distribution throughout the cross-section of the different samples for a center line on the upper sheet (0.75 mm from the surface). Several profiles have been plotted—including for the bottom sheet—and they all presented similar behaviors. 

The base material reached approximately 110 HV. When transitioning from the BM to the HAZ/TMAZ, the microhardness bottomed out quickly to a minimum of around 70 HV, before rising again in the SZ to about 80 HV. The shoulder and the probe rotated at a speed of 1500 RPM, which, according to the experiments conducted, made the temperature rise to 390 °C at one millimeter below the spot area. Due to the friction between the material and the tool, the temperature increases, which leads to a thermal softening of the material in the affected welding zones. The as-received alloy—AA6061-T6—underwent a heat treatment which introduced strengthening precipitates in the BM material. Due to the heat applied in the welded area, the precipitates were partially dissolved and coarsened, which explains the decrease of microhardness at the HAZ. In the SZ, the temperature reached its maximum and the strengthening precipitates dissolved, forming a supersaturated solution and resulting in lower hardness values in comparison to the base material. However, a slight increment in microhardness was observed in the SZ center, as shown in Figure 18. There are currently two accepted theories explaining this behavior on precipitation-hardenable aluminum alloys that are welded by RFSSW: precipitate strengthening and grain refinement. Li et al. [44] studied the rapid cooling of AA2A12-T4 alloys by TEM. The authors showed that the formed coherent S-precipitates were responsible for the locally increased hardness at the SZ. Moreover, the high process temperature and shear rate, that were created by the stirring effect of the shoulder, induces dynamic recrystallization, leading to refined grains in the SZ, thereby increasing the hardness by the Hall-Petch effect, as demonstrated in [11] for the RFSSW of AA7075-T6. Further investigations using transmission electron microscopy and electron backscatter diffraction would be needed to support the understanding of these assumptions. However, these are out of the scope of this study.

The microhardness profiles represent the microstructural changes that were not detectable under OM between the different zones. When comparing the microhardness profiles shown in Figure 18, it is possible to observe that there is no visible difference between the profiles that were obtained in samples produced by a new tool and those produced after 2500 spots. This indicates that they had experienced a similar energy input, with a low deviation. However, when combining this information with the observed hook height and measured temperature, it is possible to confirm that the heat input after 2500 spots was slightly lower than that from a new tool, since it showed a smaller hook and a lower process temperature than the new tool. On the contrary, the profile in the HAZ/TMAZ-SZ volume that was obtained after 1250 spots is slightly wider than the other profiles, which implies that the energy input was higher than that of the other analyzed specimens. This result is in accordance with the higher hook height that was observed for this condition, since a higher energy input means a higher material plasticization.

Based on the obtained results, it is possible to affirm that tool wear modifies the tool geometry and affects the energy input—i.e., the heat generation—during the weld. Hence, it influences the degree of plasticization reached by the material. This behavior is in accordance with what has already been reported for the friction-based welding processes of aluminum alloys for threaded probe tools [22,23,25,57]. Furthermore, this distinct heat input affects the hook height, which results in a change in the joint fracture mode, explaining the decrease of the ULSF with the advancing tool wear. The study of the influence of the changes in tool geometry on the metal plasticizing and material flow regimes, as well as their direct correlation on frictional heat generation, is a topic outside of the scope of this manuscript. Nevertheless, this is a fundamental topic that requires further study to fully understand and predict the final mechanical performance of friction spot welds. Experimental and modeling work are still required to fully solve and confirm the assumptions proposed in this manuscript.

## 4. Conclusions

The present work investigated the influence of RFSSW tool wear on the mechanical performance of AA6061-T6 similar welds. Based on the experimental results, the following conclusions can be drawn:

- The RFSSW process parameters were optimized for AA6061-T6 through a Box-Behnken design of experiments (BBD) and one-factor-at-a-time (OFAT) approaches, for the purpose of maximizing the obtained ultimate lap shear force (ULSF) response. The optimized parameter set—a plunge depth (DP) of 2 mm, a rotational speed (RS) of 1500 RPM and, a dwell time (DT)of 0 s—produced joints with an average ULSF of 8.45 ± 0.08 kN.
- Considerable tool wear was observed along the thread groove profile of the shoulder component, due to the series of the welds produced. Consequently, an accentuated decline of the measured yielded ULSF was observed. In addition, the wear increased the gaps between the components of the tool, which led to an accumulation of material between the shoulder and the clamping ring. This aggregation hampered the tool rotation and, therefore, the maximum torque of the machine was often reached, and the process had to be interrupted. Consequently, it is possible to confirm that the maximum number of spots capable of being produced by the tool, using the set of variables here specified is, currently, at least 2500 welds, without severely compromising the quality of such spot welds.
- A change in the fracture mode was identified. Initially, only shear-plug fracture was observed in the tested samples. However, after 2500 spots, a transition from complete shear-plug to a mixture between the shear-plug and the plug pull-out failure modes was observed. This behavior was assessed as the main cause of the ULSF marked decline.
- The microstructural characterization showed a similar average grain size for all the weld zones in the analyzed conditions, while the change in hook height indicated variations in the process heat input. Temperature and microhardness measurements were conducted to confirm this, and these showed that the tool wear affects the energy input that is delivered by the RFSSW process to the materials.
- The achieved results show that, although RFSSW is an interesting alternative for the welding of lightweight alloys, premature tool wear can highly influence the mechanical performance of the produced joints. In order to overcome this challenge, and decrease the associated process costs, a frictional heat input monitoring system must be applied which aims to keep the energy and the ULSF stable and, consequently, improves the tool lifespan. Further investigations in this direction could lead to great opportunities to improve the RFSSW process, since a constant heat input is expected to be possible if the wear is compensated by a continuous increment of either the PD or RS.

## Figures and Tables

**Table 1:** Chemical composition and mechanical properties of AA6061-T6.

Chemical Composition (wt.%)									Mechanical Properties *		
Al	Mg	Si	Fe	Cu	Cr	Zn	Mn	Ti	Tensile Strength [MPa]	Elongation [%]	Hardness [HV]
Bal.	0.8–1.2	0.4–0.8	0.7	0.15–0.4	0.04–0.35	0.25	0.15	0.15	310	12	110

* Experimental values.

**Table 2:** Welding process parameters and their selected levels.

Parameter	Abbreviation	Level Low (−1)	Level Middle (0)	Level High (1)
Rotational speed [RPM]	RS	1000	1750	2500
Dwell time [s]	DT	0	1	2
Plunge depth [mm]	PD	1.3	1.65	2

**Table 3:** Box-Behnken Design parameter matrix and ULSF experimental results.

Sample Nr.	RS [RPM]	DT [s]	PD [mm]	ULSF [kN]
1	1000	0	1.65	7.3
2	2500	0	1.65	8.0
3	1000	2	1.65	7.1
4	2500	2	1.65	7.7
5	1000	1	1.30	5.5
6	2500	1	1.30	6.5
7	1000	1	2.00	8.1
8	2500	1	2.00	7.3
9	1750	0	1.30	6.0
10	1750	2	1.30	6.5
11	1750	0	2.00	8.3
12	1750	2	2.00	8.1
13	1750	1	1.65	7.8
14	1750	1	1.65	7.6
15	1750	1	1.65	7.6
16	1750	1	1.65	7.4
17	1750	1	1.65	7.8
18	1750	1	1.65	7.7

**Table 4:** Statistically significant terms for ULSF and their respective *p*-values.

Parameter	*p*-Value
PD	<0.001
PD*PD	<0.001
RS*PD	0.002
RS*RS	0.029
RS	0.035

**Table 5:** Grain size results for a new tool, after 1250 spots, and after 2500 spots in the different zones.

	Condition	Average Grain Diameter [µm]
BM	New tool	29 ± 14
	1250 spots	25 ± 9
	2500 spots	30 ± 8
HAZ	New tool	10 ± 4
	1250 spots	9 ± 4
	2500 spots	10 ± 5
SZ	New tool	8 ± 3
	1250 spots	7 ± 3
	2500 spots	9 ± 3

## Data Availability

Not applicable.
